# Clinical and Electrophysiological Changes in Pediatric Spinal Muscular Atrophy after 2 Years of Nusinersen Treatment

**DOI:** 10.3390/pharmaceutics14102074

**Published:** 2022-09-29

**Authors:** Mihaela Axente, Andrada Mirea, Corina Sporea, Liliana Pădure, Cristina Manuela Drăgoi, Alina Crenguța Nicolae, Daniela Adriana Ion

**Affiliations:** 1 Faculty of Medicine, University of Medicine and Pharmacy “Carol Davila”, 37 Dionisie Lupu Street, 020021 Bucharest, Romania; 2 National University Center for Children Neurorehabilitation “Dr. Nicolae Robanescu”, 44 Dumitru Minca Street, 041408 Bucharest, Romania; 3 Faculty of Midwifery and Nursing, University of Medicine and Pharmacy “Carol Davila”, 37 Dionisie Lupu Street, 020021 Bucharest, Romania; 4 Department of Biochemistry, Faculty of Pharmacy, “Carol Davila” University of Medicine and Pharmacy, 020956 Bucharest, Romania; 5 Department of Pathophysiology, National Institute for Infectious Diseases Prof. Dr. Matei Balș, Carol Davila University of Medicine and Pharmacy, 1 Calistrat Grozovici Street, 021105 Bucharest, Romania

**Keywords:** spinal muscular atrophy, compound motor action potential, the children’s hospital of Philadelphia infant test of neuromuscular disorders, hammersmith functional motor scale expanded

## Abstract

In the new therapeutic era, disease-modifying treatment (nusinersen) has changed the natural evolution of spinal muscular atrophy (SMA), creating new phenotypes. The main purpose of the retrospective observational study was to explore changes in clinical evolution and electrophysiological data after 2 years of nusinersen treatment. We assessed distal compound motor action potential (CMAP) on the ulnar nerve and motor abilities in 34 SMA patients, aged between 1 and 16 years old, under nusinersen treatment, using specific motor scales for types 1, 2 and 3. The evaluations were performed at treatment initiation and 26 months later. There were registered increased values for CMAP amplitudes after 2 years of nusinersen, significantly correlated with motor function evolution in SMA type 1 patients (*p* < 0.005, r = 0.667). In total, 45% of non-sitters became sitters and 25% of sitters became walkers. For SMA types 1 and 2, the age at the treatment initialization is highly significant (*p* < 0.0001) and correlated with treatment yield. A strong negative correlation (r = −0.633) was observed for SMA type 1 and a very strong negative correlation (r = −0.813) for SMA type 2. In treated SMA cases, the distal amplitude of the CMAP and motor functional scales are important prognostic factors, and early diagnosis and treatment are essential for a better outcome.

## 1. Introduction

Spinal muscular atrophy (SMA) is a degenerative neuromuscular genetic disorder with autosomal recessive transmission characterized by progressive loss of spinal and brainstem motor neurons with severe hypotonia, muscle weakness, atrophy, swallowing, and respiratory dysfunction [1,2]. 

Weakness is predominantly in the girdles and truncal, with greater involvement of the lower limbs and areflexia on examination. Bulbar and respiratory muscle weakness can occur in infantile type 1, particularly in more severe cases. Facial and ocular muscles are generally spared [3].

About 95% of the cases result from decreased amounts of survival motor neuron (SMN) protein due to deletions or mutations in the SMN1 gene, on chromosome 5q. 

Most cases of SMA (95%) are caused by the biallelic homozygous deletion mutation in the SMN1 gene at 5q [4], with subsequently decreased amounts of survival motor neuron (SMN) protein. There are rare situations with compound heterozygous variants or punctiform mutations. The allelic SMN2 gene, also present on chromosome 5, contributes to a small extent to the production of SMN protein and thus to the phenotypic aspect of the disease [5]. The SMN2 gene differs from the SMN1 gene by only five nucleotides. The substitution cytosine-thymine in the sixth position of exon 7 disturbs splicing, resulting in exon 7 exclusion and a rapid degradation of SMN2 protein. The SMN2 gene produces up to 10% of full-length SMN protein due to normal splicing. The number of copies of the SMN2 gene varies from zero to eight, correlating directly with the amount of functional SMN protein and predicting disease severity. 

Antisense oligonucleotides therapy of SMA is directed towards increasing the inclusion of exon 7 in the mRNA of the SMN2 gene to increase functional SMN protein [6]. 

SMA includes a wide range of phenotypes that are classified into clinical groups based on onset and maximum motor function achieved (untreated patients): very weak infants unable to sit unsupported (type 1) [7]; non-ambulant patients able to sit independently, and even walk with support (type 2); ambulant patients, with normal neurological development during early childhood, but with progressive loss of muscle strength (type 3) [8]. Approximately 95% of SMA type 1 patients have two SMN2 gene copies; 80% of SMA type 2 patients have three copies and 97% of type 3 patients have three to four copies. Recent recommendations [6] subdivided the functional classification from the original consensus statement document into three groups: non-sitters, sitters, and walkers.

### 1.1. Electrophysiology

CMAP represents motor fiber action potential summation in a motor area. It is obtained by the supramaximal stimulation of a peripheral motor nerve [9]. It is easy to obtain but a non-specific parameter, which does not provide enough information about chronic reinnervation mechanisms. CMAP amplitude decrease is observed in most of the symptomatic patients, as SMA is a degenerative peripheral motoneuron disease. Nevertheless, due to compensatory changes (collateral reinnervation), CMAP amplitude can be maintained. With the development of innovative therapies, in clinical trials, CMAP was introduced as a monitoring parameter and prognostic factor of the patients’ evolution [10]. A sharp decrease in it was also noticed with the installation of motor regression, especially in the first form of SMA [11].

### 1.2. Treatment

The development of nusinersen, a synthetic, stabilized antisense oligonucleotide (ASO) that targets the splicing of SMN2 and increases the formation of a more stable protein product, has transformed the fatal type of SMA (type 1) into a stable condition. The first approved SMN2 pre-mRNA targeted therapy consisted of modified ASO, which was designed to bind to the intronic splice silencer site located in intron 7 of SMN-2 pre-messenger RNA, promoting exon 7 inclusion at the SMN2 messenger RNA level [12,13,14,15]. Earlier treatment leads to better outcomes, and initiation before symptom onset is associated with nearly normal early motor development in some infants [16].

In Romania, the first nusinersen administration was in October 2018 in the National University Center for Children Neurorehabilitation “Dr. Nicolae Robanescu”. This procedure followed all the legal regulations for the patients’ safety [17].

Table 1 shows the functional scales used based on the patient’s age, SMA type, and neurological condition. Examinations using scales should be carried out by physiotherapists or physicians, employing tests based on the standard neurological examination [18]. 

The primary study objective was to examine the relationship between clinical evolution and electrophysiological data in SMA patients after two years of nusinersen treatment, investigating the distal ulnar CMAP amplitude and assessing CHOP/HFMSE/6MWT score at nusinersen initiation (T0) and every 4 months up to 26 months later (T26). Our working hypothesis was that there are statistically relevant correlations between clinical and electrophysiological data after 2 years of nusinersen in our cohort of SMA patients.

### 1.3. Ethical Approval

The study was conducted according to the guidelines of the Declaration of Helsinki and approved by the Ethics Committee of National University Center for Children Neurorehabilitation “Dr. Nicolae Robanescu” No. 7465/01.10.2018. Data were collected during periodic evaluations, in accordance with our drug administration protocols, and all patients’ parents signed informed consents.

## 2. Materials and Methods

We assessed motor abilities in 34 SMA patients diagnosed with types 1, 2, and 3, aged between 1 and 16 years old. 33 patients had biallelic deletion of SMN1 and 1 compound heterozygous, with 2 or 3 SMN2 copies, under nusinersen treatment. The assessments were performed at treatment initiation (T0) and every 4 months up to 26 months (T26). Assessments were performed at nusinersen administration time (Injection 1–Injection 10) using The Children’s Hospital of Philadelphia Infant Test of Neuromuscular Disorders (CHOP INTEND) for type 1 and Hammersmith Functional Motor Scale Expanded (HFMSE) for SMA types 2 and 3. Simultaneously, the distal CMAP on the ulnar nerve was recorded. The study was carried out between October 2018 and October 2021.

Patients with genetic confirmation of SMA, pediatric age (0–18 years old) and 2 or more copies of SMN2 were included in the study. 

Patients who were currently receiving another medication (risdiplam or onasemnogene abeparvovec-xioi) or who were agitated or uncooperative were excluded.

CMAP was recorded in the distal ulnar nerve, by supramaximal stimulation. Electrophysiological examinations were conducted using a 6-channel EMG Keypoint. No sedation was administered. According to good standing practice [19], a pleasant atmosphere was created to ensure the child’s and the family’s relaxation to facilitate the best possible approach. The recorded distal skin temperature was between 36.5 and 37 degrees in all children. We used surface pediatric electrodes placed on the hypothenar eminence. The electrical stimulation was performed distally (wrist) on the ulnar nerve, using progressive intensities ranged between 10 and 90 mA to obtain maximal amplitude. The CMAP was feasible and reproductible (electrical stimulation was repeated 3 times). 

CHOP INTEND was developed to assess the motor abilities of patients with SMA type 1 before the age of six months who never achieve independent sitting [20,21,22,23]. Each of the 16 items on this scale is graded on a scale from 0 (no response) to 4, with 0 being the lowest grade and 4 being the highest (complete response). The total number of possible points ranges from 0 to 64.

HFMSE allows for the assessment of high-functioning SMA type 2 and includes 20 items from the Hammersmith Functional Motor Scale (a tool for evaluation of motor function in pediatric patients with SMA type 2, not designed for ambulatory patients), and 13 items from Gross Motor Function Measure. HFMSE can distinguish between ambulatory SMA type 3 patients and allows for the inclusion of a broader range of intermediate and mild SMA patients [23,24,25,26,27]. The score ranges between 0 and 66.

A 6 min walk test (6 MWT) [28] is a submaximal exercise test used to assess aerobic capacity and endurance. The distance covered over 6 min is used as the outcome by which to compare changes in performance capacity.

The percentage yields for CHOP/HFMSE scores’ evolution were calculated based on SMA type. The percent yield is calculated by multiplying the actual yield (the actual improvement in motor development after 26 months of nusinersen treatment) by the theoretical yield (the maximum possible value of motor development).

Simultaneously, we tracked the evolution of CMAP under treatment at the same time intervals, 26 months.

The statistical data processing was performed using the Statistical Package for the Social Sciences IBM SPSS Statistics 24 and Excel 2021 software. The data processing for the analysis of the potential correlations between the collected data followed-up on two statistical indicators: statistical significance (*p*) and Pearson correlation coefficient (r) [29,30]. The Pearson correlation coefficient is a measure of linear correlation between two sets of data. It returns a value between −1 and 1, where 1 indicates a strong positive relationship, 0 indicates no relationship at all, and −1 indicates a strong negative relationship. The r value indicates a moderate correlation if 0.40 < r < 0.70, a strong correlation if 0.70 < r < 0.90 and a very strong one if r > 0.90.

## 3. Results

At the start of treatment (T0), the SMA type I group consisted of 11 non-sitter patients. After 10 administrations of nusinersen (T26), 45.45% became sitters (Table 2), with their CMAP amplitude increasing from 0.53 ± 0.23 to 1.85 ± 1.05. The other 54.55% patients who remained non-sitters showed an increase in CMAP from 0.26 ± 0.23 to 1.19 ± 0.66. 

At T0, the type II SMA group consisted of 16 sitters. At T26, 25% of the patients became walkers (Table 2), with their CMAP amplitude increasing from 2.73 ± 2.08 to 3.65 ± 1.55. The other 75% of patients who remained sitters showed an increase in CMAP from 0.99 ± 0.91 to 1.73 ± 1.18. 

The SMA type III group consisted of four sitters and three walkers, both at T0 and T26 (Table 2). The CMAP of the sitters increased from 1.25 ± 0.55 to 2.05 ± 0.66, while the CMAP of the walkers increased from 2.73 ± 2.38 to 3.66 ± 2.85.

**Figure 1:**
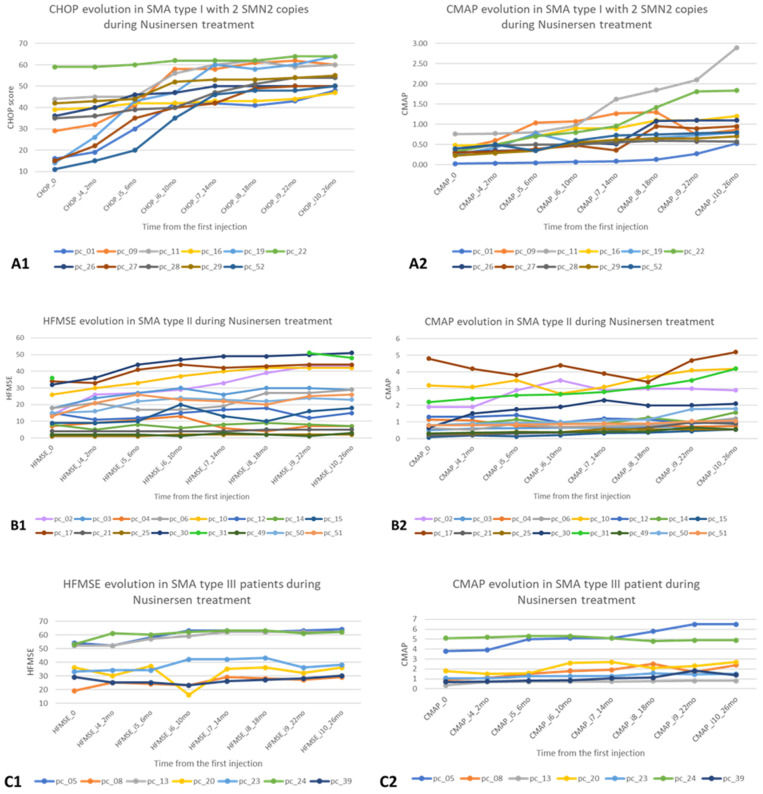
Motor and electrophysiological evolution in the first 26 months after starting nusinersen treatment for patients with SMA types 1, 2 and 3. (**A1**)—CHOP evolution in SMA type 1 with 2 copies of SMN; (**A2**)—CMAP evolution in SMA type 1 with 2 copies of SMN; (**B1**)—HFMSE evolution in SMA type 2; (**B2**)—CMAP evolution in SMA type 2; (**C1**)—HFMSE evolution in SMA type 3; (**C2**)—CMAP evolution in SMA type 3.

Figure 1 presents the motor and electrophysiological changes in the first 26 months after starting nusinersen treatment for each patient with SMA types 1, 2 and 3.

There is a significant increase in CHOP, consistent with CMAP in 5 patients for SMA type 1. In SMA type 2 patients, HFMSE is generally stationary, with only two of them having significant variations in CMAP (above the group average). In SMA type 3 patients, CMAP/HFMSE show a stationary trend between T0 and T26.

**Figure 2:**
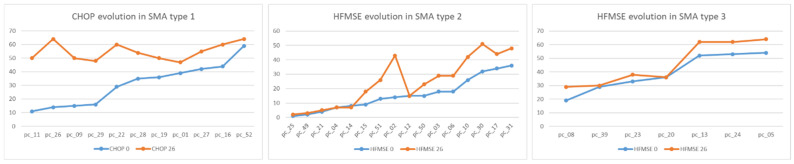
CHOP and HFMSE evolution at T0 and T26 for each type of SMA.

Motor function evolution after 26 months of nusinersen treatment is shown in Figure 2.

In SMA type 1, we observed CHOP increase between T0 and T26. In SMA type 2, there were two patients with a better outcome than the others who had differences between T0 and T26, and whose evaluations slightly increased. In SMA type 3, no regression was registered, but very close values were obtained in both evaluations, with a single patient having the same HFMSE score at T0/T26.

**Figure 3:**
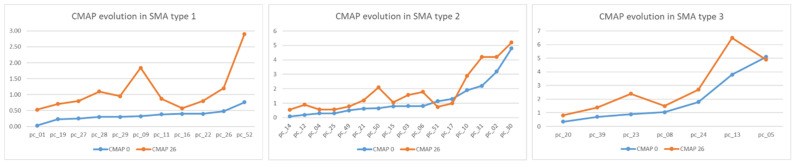
CMAP evolution at T26 for each type of SMA.

Electrophysiological changes in ulnar nerve conduction at T0 and T26 are illustrated in Figure 3.

The most significant increase in distal CMAP amplitude was recorded in SMA type 1 patients, statistically correlated with scores on CHOP scales (*p* < 0.001). 

Most of the SMA type 2 patients started treatment long after symptom onset (13–96 months) and presented stationary CMAP amplitude and HFMSE scores. 

The majority (four out of seven patients) of SMA type 3 patients were non-ambulant at T0. They started treatment several years after losing gait, remaining non-ambulant at T26.

Low values of CMAP amplitude at the distal ulnar nerve were observed. CMAP amplitude is shown to be significantly (*p* < 0.005) strongly correlated (r = 0.667) with motor function evolution only in SMA type 1. For SMA types 2 and 3 there are no significant statistical correlations.

## 4. Discussion

According to the obtained results, there is a correlation between the increase in the score on the functional scales and an increase in CMAP in patients with SMA type 1 who have passed into a higher motor stage; this is correlated with the early start of treatment (shortly after the onset of motor regression). 

Patients who were diagnosed a few months after the loss of motor acquisitions regained them in the first months after starting treatment. Additionally, the children with borderline shapes passed in superior form, with some acquiring the sitting position and others assisted/independent walking. The favorable evolution depends on a complex of factors—some proven (compliance with standards of care—physical therapy [31], respiratory nursing [32], early treatment of secondary scoliosis [33,34]), others still under research— such as genetic factors (number of SMN2 copies or other yet undiscovered aspects) and biochemical factors (level of cerebrospinal fluid neurofilaments). 

Patients who had a higher amplitude of CMAP (number of motoneurons/axons) at the beginning of treatment had a better clinical evolution, thus demonstrating that CMAP is a marker of the degenerative process.

We had a particular case, a compound heterozygous case, aged 9 years, who started treatment with nusinersen at the age of 7. In particular, there was a discrepancy between motor level (ambulatory) and extremely low level (<1 mV) of CMAP amplitude at the level of the ulnar nerve, which increased slightly during 2 years of treatment. Further research is needed on biomarkers (genetic, biochemical) to investigate particular clinical and electrophysiological responses in each case.

A relevant correlation was found between the number of SMN2 copies and the disease phenotype, with patients with fewer SMN2 copies having a more modest clinical evolution and an extremely low CMAP.

Nusinersen treatment has changed the paradigm of this disease evolution; early forms with neonatal onset or in the first months of life, with severe bulbar dysfunction, are no longer fatal, and in late forms have significantly improved motor performance and quality of life. We found a clinically significant improvement in patients who started treatment as soon as possible [35] at the time of the first signs of the disease (motor regression), both in the early and late forms. 

## 5. Conclusions

After 2 years of nusinersen treatment, children with SMA types 1, 2 and 3 had a favorable clinical evolution, as shown by the scores on the motor scales. Clinical data were correlated with electrophysiological data, with statistical significance in SMA type 1. However, it is important to follow up CMAP as an electrophysiological marker to capture motor regression [36], but also as a prognostic factor. 

## Figures and Tables

**Table 1:** Recommended functional scales according to patient’s functional status.

Patient’s Functional Status	Recommended Functional Scales
Non-sitter	CHOP INTEND
Sitter	HFMSE
Walker	6 MWT, HFMSE

**Table 2:** Clinical data before and after 26 months of nusinersen in SMA type 1, 2, and 3.

Variables	Non-Sitters		Sitters		Walkers	
Time	T0	T26	T0	T26	T0	T26
Age at treatment initiation (months)	2–76		13–196		30–185	
SMA subtype						
- SMA 1	11 (100%)	6 (54.55%)		5 (45.45%)		
- SMA 2			16 (100%)	12 (75%)		4 (25%)
- SMA 3			4 (57.14%)	4 (57.14%)	3 (42.8%)	3 (42.8%)
SMN2 copies						
- 2 copies	11 (100%)		3 (18.75%)		2 (28.57%)	
- 3 copies			13 (81.25%)		5 (71.43%)	

## Data Availability

The data presented in this study are available on request from the corresponding author.
